# Implementation Factors of Digital Health Interventions in Depression Care—The Perspective of Health Professionals

**DOI:** 10.3390/healthcare13212717

**Published:** 2025-10-27

**Authors:** Jessica Hafner, Pinar Tokgöz, Christoph Dockweiler

**Affiliations:** 1 School of Public Health, Bielefeld University, 33615 Bielefeld, Germany; 2 Department of Social Sciences, Faculty of Arts and Humanities, University of Siegen, 57076 Siegen, Germany; pinar.tokgoez@uni-siegen.de (P.T.); christoph.dockweiler@uni-siegen.de (C.D.)

**Keywords:** digital health interventions, depression, mental health, CFIR, implementation, qualitative research

## Abstract

**Background/Objectives**: Depressive disorders are among the most prevalent mental illnesses worldwide. Digital health interventions offer potential to improve access, efficiency, and outcomes in depression care. However, their sustainable integration into routine clinical practice remains limited. This study explored individual, organizational, external and contextual factors influencing digital health interventions implementation from the perspective of health professionals. **Methods**: Semi-structured interviews with health professionals (*n* = 9) were analyzed using a hybrid qualitative approach. First, structuring content analysis following Kuckartz was applied to systematically code and categorize the transcripts. Second, the resulting codes were mapped onto four domains of the Consolidated Framework for Implementation Research (Outer Setting, Inner Setting, Process, and Characteristics of Individuals) to identify implementation-relevant barriers and facilitators. This combined approach ensured a transparent, theory-informed, and reproducible analysis of factors influencing digital health intervention implementation in depression care. **Results**: Key individual-level enablers included openness to innovation, motivation, and prior experience with digital tools. Organizational factors such as leadership support, designated facilitators, time, training, and IT infrastructure were critical. External factors included data protection, clear regulatory frameworks, reimbursement mechanisms, and scientific validation. Barriers involved limited digital skills, ambiguous responsibilities, and concerns about misuse or risks. **Conclusions**: The successful implementation of digital health interventions in depression care requires alignment with organizational structures, provider capabilities, and patient needs. Supportive leadership, tailored training, and clear external frameworks can enhance acceptance and sustainability. As complementary tools, digital health interventions can help optimize mental health services and improve patient outcomes.

## 1. Introduction

Depressive disorders are among the most common mental illnesses worldwide and represent a major global health concern [1]. In Germany, administrative data indicate that 16.7% of adults registered in health records received a diagnosis of depression in 2023 [2]. These disorders are associated with chronic courses, frequent comorbidities, and substantial impairments in patients’ quality of life, which also affect their relatives [3,4,5]. They generate high healthcare costs and indirect costs from reduced work capacity and premature retirement, placing pressure on the healthcare system [6]. Access to care is further limited by long waiting times [3], restricted multiprofessional services [7], and personal barriers such as time constraints and stigma [8], which further widen the care gap [9].

In response to these challenges, there has been increasing momentum—nationally and internationally—for the development of digitally supported care models in the field of mental health [10]. A growing body of empirical research confirms the effectiveness of digital health interventions (DHIs), particularly in reducing symptoms, improving quality of life, and decreasing healthcare costs [11,12]. DHIs hold considerable promise for mitigating access barriers and enhancing care structures [3].

For sustainable use, however, DHIs must be adapted to user needs and organizational contexts, ideally through participatory development [13].

As implementation is influenced by prevailing social and cultural norms, which are deeply contextual and ever changing, it is important to consider organizational circumstances when adopting DHIs for depression care [14]. Existing studies provide only limited insights into the facilitators and barriers of implementation—especially concerning depressive disorders. Most research focuses on user acceptance [15], while organizational conditions receive comparatively little attention [16,17]. For instance health professionals (HPs) struggle to change established clinical practices required to implement DHIs without adequate leadership support [18]. Moreover, regulatory and reimbursement challenges may further hinder the integration of DHIs into healthcare systems, making it difficult for HPs to offer these services [19,20,21]. A deeper understanding of usage patterns, professional experiences, and organizational requirements is needed to ensure effective and lasting integration of DHIs [22,23]. Despite their demonstrated effectiveness, many projects fail to achieve sustained implementation and broader dissemination in routine practice [24].

To address these gaps, the present study investigates the individual, organizational, external, and contextual factors that influence the implementation of DHIs in depression care, as perceived by health professionals (HPs).

## 2. Materials and Methods

### 2.1. Study Design

Given the limited empirical knowledge in this area, an inductive qualitative design was employed to capture participants’ perspectives and explore the meanings of their experiences [25]. Semi-structured face-to-face interviews with open-ended questions were conducted to gather detailed accounts of participants’ experiences with DHIs and their interpretations of social reality.

To systematically investigate the factors influencing the organizational implementation of DHIs in depression care, this study draws upon the Consolidated Framework for Implementation Research (CFIR). The CFIR provides a comprehensive framework for understanding factors that influence implementation across multiple levels of the healthcare system.

The CFIR comprises five interrelated domains that collectively shape the implementation of an intervention. The implementation process is fundamentally influenced by the intervention’s intrinsic characteristics (domain 1: Intervention). This process is situated within the internal organizational context (domain 2: Inner Setting) and shaped by the individuals involved, including their roles, attitudes, and interactions (domain 3: Characteristics of individuals). Furthermore, the broader external context (domain 4: Outer Setting), encompassing societal, cultural, and political determinants, plays a critical role in modulating the implementation. These domains interact dynamically throughout the stages of the implementation process itself (domain 5: Process), which includes planning, execution, reflection, and evaluation [26,27]. This article follows the Standards for Reporting Qualitative Research [28] (see Supplementary Material S1: Standards for Reporting Qualitative Research).

### 2.2. Participants

Participants were required to meet the following criteria: (1) HPs had to be 18 years of age or older, (2) HPs had to be actively engaged in the care of people with depression and (3) HPs had to belong to one of the following professional groups: specialist in psychiatry, specialist in psychotherapy, medical and psychological psychotherapist, advanced practice psychiatric nurse and general practitioner. All participants were HPs currently working in outpatient and inpatient mental healthcare settings, including general practitioners in outpatient private practice. Participants were from Germany.

Recruitment was conducted using a snowball sampling method. For this purpose, target group-specific informational materials were developed, and informational interviews were conducted. Additionally, a selective case sampling was performed in accordance with the inclusion criteria. Recruitment took place between February and July 2025 using a snowball approach combined with selective case sampling. The initial recruitment base comprised professional and institutional networks in psychiatric and psychotherapeutic care in Germany. Study information sheets and invitation letters were distributed in hospitals, psychotherapeutic and general medical practices. In addition, heads of departments and research units in relevant institutions were contacted via e-mail and telephone. University-based professional contacts and mailing lists were also used to reach potential participants.

No financial or material incentives were provided. Participation was voluntary and based on written informed consent. The exact number of individuals contacted cannot be specified because several overlapping channels were used. According to feedback from non-participants, the most frequent reason for declining participation was lack of time due to high clinical workload. No prior professional or supervisory relationships existed between the researchers and the participants.

Participants were recruited using a theoretical sampling approach until theoretical saturation was reached. This ensured that data collection continued until no new themes or relevant implementation factors emerged. Consequently, the identified implementation determinants provide a comprehensive reflection of the diversity of perspectives held by HPs.

### 2.3. Data Collection

The interview guide covered three thematic areas. The first complex included a general introduction to the subject area in order to clarify the necessary terminology. The second complex was structured according to the CFIR, including questions regarding organizational implementation conditions for depression care. The third complex addressed follow-up questions, both immanent and exmanent. To test the comprehensibility of the interview guide and identify potential problems in answering the questions, two cognitive pre-tests were conducted with HPs. Example questions from the original interview guide included: (1) “From your perspective, what role does policy play in the implementation of the intervention into routine care?” and (2) “To what extent do you think leadership personnel are responsible for specific functions and tasks?” Based on their feedback, several revisions were made to improve clarity and reduce redundancy. One of the main points of criticism concerned the overall length of the interview. As a result, the guide was shortened by merging overlapping questions and removing less relevant items. The semi-structured interview guide used in this study is provided in the Supplementary Materials (Supplementary Material S2: Semi-Structured Interview Guide).

Additional socio-demographic characteristics were collected and affinity for technology interaction (ATI) was calculated to better describe the sample and identify possible correlations. The ATI was surveyed using the 9-item affinity for technology interaction scale, which is designed to assess a person’s tendency to actively engage in intensive technology interaction [29]. The affinity for technology can have a value between 1 and 6, with 6 being the highest value. The questionnaire applied in this study is provided for reference (Supplementary Material S3: Affinity for Technology Questionnaire).

Between April and July 2025, nine semi-structured interviews were conducted, lasting on average 42 min (range: 34–51). All interviews were conducted face-to-face or via videoconference in German and subsequently translated into English. The translations were carried out carefully using a combination of the researchers’ personal language proficiency, language-support software (Grammarly, https://www.grammarly.com/, accessed on 15 September 2025; Grammarly Inc., San Francisco, CA, USA), and review by a scientific expert to ensure accuracy and consistency. 

### 2.4. Data Analysis

All interviews were transcribed verbatim by the authors. Data were analyzed using a hybrid qualitative approach, combining structuring content analysis according to Kuckartz [30] with CFIR-guided thematic mapping. This approach allowed systematic coding of transcripts while linking findings to implementation-relevant domains. First, transcripts were organized and coded deductively according to the predefined categories of the interview guide. Second, participants’ statements were mapped to the CFIR domains and further refined through inductive coding to capture emerging, context-specific determinants. Third, categories were compared across participants by grouping similar themes and synthesizing overarching patterns.

The coding process was conducted using Microsoft Excel, version 2509 (Microsoft Corp., Redmond, WA, USA). Two researchers independently coded the material in two iterative coding cycles. Discrepancies were discussed in joint sessions until full consensus was reached. Although no formal intercoder reliability coefficient was calculated, the iterative discussion ensured interpretative consistency and analytical transparency. A structured category system was developed, combining CFIR-informed and inductively derived categories, and was continuously refined throughout the analysis process.

The analysis focused on four CFIR domains: Outer Setting, Inner Setting, Process, and Characteristics of Individuals. The Intervention domain was deliberately excluded from the data collection and analysis. This decision was based on two considerations: (1) addressing all CFIR domains in interviews substantially extended duration and reduced participant engagement, and (2) the primary analytical focus of this study was on organizational, external and contextual factors influencing implementation. Intervention-related determinants had already been examined in prior acceptance studies and would have exceeded the scope of the present research.

**Figure 1:**
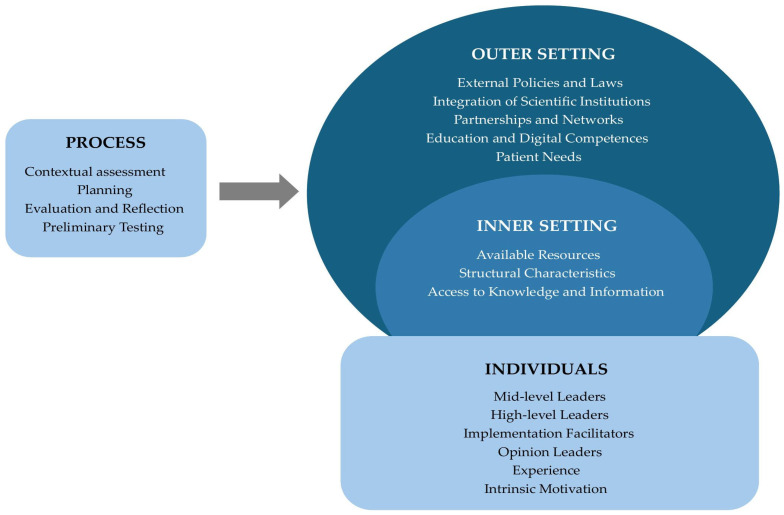
Modified CFIR model used for data analysis, including four domains (Outer Setting, Inner Setting, Process, and Individuals).

It should be noted that the resulting representation reflects a modified version of the CFIR. In contrast to the original CFIR, which consists of five domains, this adapted version includes four domains—Outer Setting, Inner Setting, Process, and Characteristics of Individuals—while the Intervention domain was excluded. This adaptation reflects the study’s specific focus on organizational, external and contextual factors influencing the implementation of DHIs for depression care. Figure 1 illustrates the adapted CFIR framework applied in this study. To enhance methodological transparency, the CFIR-informed category system and the analytical codebook are presented in the Supplementary Materials (Supplementary Table S1: CFIR-informed Category System; Supplementary Table S2: Analytical Codebook).

Although the Intervention domain was not explicitly included as a separate analytical dimension, many of its constructs emerged naturally within the data. Participants frequently discussed issues such as intervention adaptability, usability, cost, and perceived evidence strength. These aspects were therefore integrated inductively into other CFIR-informed categories. Accordingly, the present analysis can be best described as CFIR-informed, reflecting an adapted framework that combines deductive CFIR guidance with inductive openness to emergent data.

The research team received training in qualitative research methods, including content analysis and thematic coding, and had prior experience conducting semi-structured interviews. During data collection and analysis, the team continuously reflected on their own perspectives, assumptions, and potential biases that could influence interpretation. Reflexive discussions were held regularly to ensure analytical rigor, consensus in coding decisions, and transparency in the development of themes. Any discrepancies or differences in interpretation were discussed until agreement was reached.

This reflexive process helped mitigate the influence of individual preconceptions and strengthened the credibility and trustworthiness of the study findings.

### 2.5. Ethical Considerations

The study was approved by the Ethics Committee of the University of Bielefeld (EUB-2022-144-S). All participants were informed about the study objective and the intended use of the interview data. All subjects gave their informed consent for inclusion before they participated in the study. To ensure anonymity, participants were assigned numerical codes.

### 2.6. Sampling

The sample consisted of nine HPs: three specialists in psychotherapy, two medical and psychological psychotherapists, one specialist in psychiatry, one advanced practice psychiatric nurse, one general practitioner, and one participant who preferred not to disclose professional details. Of the nine participants, eight were women and one was a man. Two respondents were between 21 and 39 years of age, three were over 40 years old, and three were older than 50 years. One participant chose not to disclose demographic details.

Regarding professional background, the sample included five licensed psychological psychotherapists for adults, one child and adolescent psychologist, one nursing specialist, one employed chief physician in psychology, and one self-employed general practitioner. In terms of employment, four participants were salaried employees, three operated private practices, and one participant combined both employment and private practice.

Participants’ affinity for technology ranged from 1.8 to 4.4 (M = 3.20, SD = 0.85), indicating a moderate overall familiarity with technology in the sample. Table 1 summarizes participant characteristics.

## 3. Results

The qualitative analysis revealed several key themes regarding the organizational implementation of DHIs from the perspective of HPs. These are presented below according to thematic categories derived from the data.

### 3.1. Individuals

HPs’ individual characteristics—such as motivation, previous experience with digital tools, and openness toward technology—emerged as central determinants shaping the adoption and sustained use of digital health interventions in depression care. Participants reported that intrinsic motivation and perceived patient benefit encouraged engagement with DHIs:

*“Well, I think it’s definitely an incentive when you feel that patients are better cared for. It’s often very frustrating—you do the initial assessment, recommend psychotherapy, but you know it’ll take forever until a spot becomes available.”*

*(P02, f, 21–29 years, inpatient mental health setting, HP)*

Eight participants had prior professional experience with DHIs. They highlighted the potential for high-frequency, location- and time-independent treatment and expressed openness toward long-term integration into clinical practice. Some participants suggested that younger professionals with higher technological affinity might be more likely to use DHIs and perceive their implementation as beneficial; however, given the small sample size (*n* = 9) and limited variance, this observation should be interpreted cautiously.

Several participants observed that patients engaging with DHIs early in their care pathway were generally better informed and prepared for therapeutic processes (*n* = 3):

*“Yes, I would like to see DHIs used more frequently. Those who had already used an App before starting therapy were much better prepared. You could start from a different point entirely.”*

*(P06, m, 50–59 years, outpatient private practice, HP)*

Five interviewees emphasized the importance of mid-level and high-level leaders for the sustainable implementation of DHIs. A smaller group stressed individual responsibility, arguing that practitioners should take initiative to explore and integrate suitable digital tools:

*“I don’t think it necessarily has to be a leadership task. Every psychotherapist should be responsible for asking themselves how they want to complement their therapy, how to structure it, and which tools to use. I do this in my own therapeutic work too—thinking about what methods to apply.”*

*(P04, f, 50–59 years, outpatient mental health setting, HP)*

A recurring theme concerned the need for designated implementation facilitators (*n* = 5). Participants indicated that operational tasks could be delegated to these facilitators, who might be supported by interdisciplinary implementation teams (*n* = 4). Furthermore, participants suggested that involving several engaged individuals may enhance the acceptance of DHIs among staff. A minority of participants (*n* = 3) recommended involving informal opinion leaders to support implementation:

*“It doesn’t have to be someone from management. It helps if there’s someone in the team that people trust and respect—someone who can advocate for new approaches without being in a formal position of power.”*

*(P09, f, 30–39 years, inpatient mental health setting, HP)*

### 3.2. Inner Setting

Within the inner organizational context, the availability of time, financial resources, technical infrastructure, and targeted training were identified as key enablers or barriers to implementation. Participants emphasized that institutional readiness and professional competence determine whether DHIs can be integrated effectively into existing workflows.

*“Technical disruptions are a major issue. I think that’s the biggest factor impacting session quality. Problems like poor audio occur regularly, and you can really feel how it breaks the flow of the session. It takes time to get back into the conversation.”*

*(P03, f, 30–39 years, inpatient mental health setting, HP*)

Participants consistently emphasized that adequate staffing, training, and IT infrastructure are critical prerequisites for the effective integration of DHIs (*n* = 8). Several participants suggested that availability of resources influences whether DHIs can be implemented successfully.

In addition to technological infrastructure, participants consistently identified time resources as essential. They noted the need to revise staffing structures to allocate sufficient time for DHI-related tasks and ensure private, undisturbed environments during implementation. Comprehensive, professionally designed training was also considered a prerequisite for clinicians to competently apply DHIs in routine care.

*“There are many different kinds of resources that are lacking—especially time and money. The financial side, the cost–benefit ratio, really needs to be evaluated.”*

*(P01, f, 40–49 years, inpatient mental health setting, HP)*

Four older respondents explicitly emphasized the importance of conducting a thorough cost–benefit analysis. Across the sample, access to professional knowledge and practical information was regarded as a fundamental prerequisite for the responsible use of DHIs (*n* = 9). Participants expressed that DHI deployment must adhere to professional standards and competence, particularly given the sensitivity of health-related data and the vulnerability of patients. Most participants reported a need for structured training sessions combining theoretical input and hands-on practice.

*“There should be lectures for therapists and physicians on different topics—just to provide basic information and orientation. Things like: how do I implement this in a practice setting? How do I integrate it into my day-to-day work?”*

*(P03, f, 30–39 years, inpatient mental health setting, HP)*

Particularly in depression treatment, some participants suggested that clinicians must be familiar with DHI functionalities and appropriate use to judge when they support treatment and when they might inadvertently reinforce maladaptive behaviors.

There was no consensus among participants regarding who should deliver DHI-related training or its frequency. A majority indicated that it would be sufficient if a designated implementation facilitator were well-trained and passed on this knowledge in a single training session (*n* = 6). In contrast, a minority—mainly older participants with lower technological affinity—favored regular training formats such as ongoing supervision or periodic refresher courses. Suggested institutions for training included the Federal Association of Contract Psychotherapists (Bundesverband der Vertragspsychotherapeuten e.V.), the State Chambers of Physicians (Ärztekammer), and the General Practitioners’ Association (Hausärzteverband). Participants overwhelmingly agreed that training content should not be provided by technology vendors or pharmaceutical representatives, to maintain scientific neutrality and ensure unbiased information (*n* = 6).

Some participants suggested that training and competence influence safe and effective adoption of DHIs. A minority emphasized that clinicians must be familiar with DHI functionalities to judge when they support treatment and when they might inadvertently reinforce maladaptive behaviors.

### 3.3. Process

The implementation process itself was perceived as most successful when supported by structured planning, clearly defined responsibilities, and opportunities for reflection. Participants emphasized that early involvement of the team, pilot testing, and internal evaluation mechanisms are essential steps to facilitate acceptance, efficiency, and sustainability. Several participants suggested that conducting a thorough contextual assessment prior to implementation helps to identify potential barriers and enablers early, supporting adoption and delivery of the innovation.

*“For many new initiatives on the ward, we have established the practice of asking staff about their satisfaction, areas where they desire changes, or where additional support might be needed. I would want to observe how the process unfolds and gauge the overall feedback—whether staff generally manage well or encounter difficulties.”*

*(P07, sex/age not disclosed, inpatient mental health setting, HP)*

Participants reported that developing a pre-established implementation plan was considered necessary to ensure a structured, accepted, and goal-oriented rollout. Some participants suggested that early involvement of all team members helps to create a sense of ownership and facilitates acceptance (*n* = 4).

A minority—especially among older participants or those with lower technological affinity—preferred a time-limited pilot phase to preliminarily test technology reliability, user-friendliness, and workflow integration (*n* = 2). These pilot phases were suggested as opportunities to make small adjustments before wider implementation.

Several participants highlighted the importance of aligning implementation efforts with available resources and specific needs of the institution. Proper allocation of time, personnel, and financial resources was seen as essential for high-quality care. Older participants and those less familiar with technology emphasized that clearly defining responsibilities and having designated contact persons in advance helps reduce uncertainty (*n* = 3).

Participants further suggested that internal evaluation and reflection mechanisms are valuable. Tools such as questionnaires, feedback discussions, and team meetings allow assessment of the implementation process, user satisfaction, and areas needing adjustment:

*“For many initiatives on our ward, we routinely collect feedback from staff and discuss what worked, what didn’t, and where additional support might be needed. This helps us adjust the process before scaling up.”*

*(P07, sex/age not disclosed, inpatient mental health setting, HP)*

Some participants suggested that these measures not only support efficiency but also reinforce staff engagement and the perception of legitimacy of the new interventions. While these perspectives reflect the views of participants rather than universal claims, they highlight perceived factors influencing successful DHI implementation.

### 3.4. Outer Setting

External conditions—including legal frameworks, data protection, reimbursement mechanisms, and scientific validation—were viewed as decisive factors for the long-term sustainability of DHIs from the perspective of the HPs. Participants highlighted the influence of political regulation, professional associations, and patient digital literacy on implementation success. Analysis of external structural conditions revealed that most participants emphasized the need for clear political and legal regulation (*n* = 7). Robust data protection and cybersecurity frameworks were considered critical, especially in mental healthcare where handling sensitive personal data is inherent to treatment. Several interviewees recommended involving independent external bodies for prior assessment of digital applications:

*“I think there’s a lot that politics can do—ideally by scaling things back or establishing an external oversight body that reviews applications beforehand. I imagine such assessments would consider data protection and the presence of indications or contraindications.”*

*(P03, f, 30–39 years, inpatient mental health setting, HP)*

Older respondents with lower technical affinity highlighted the importance of anticipating practical risk scenarios, such as third-party involvement or unauthorized access, to ensure legal protection for service providers:

*“What legal frameworks exist? What are the data protection measures in place? For example, what happens if relatives walk into the room during a video consultation, or if a therapeutic SMS is sent and someone else reads it? How is the clinic legally protected in such cases?”*

*(P08, f, 40–49 years, inpatient mental health setting, HP)*

A central structural barrier identified by participants relates to financial reimbursement mechanisms. Regardless of age or technological familiarity, there was a shared perception that national health policy must establish regulatory frameworks to ensure long-term financing of DHIs (*n* = 9). Participants emphasized the need for transparent remuneration, financial incentives, targeted investment, and funding programs. Health insurance coverage and reimbursement for individual interventions were viewed as foundational requirements. Participants suggested that financing models should align with the realities of clinical practice, for instance by incorporating standardized billing codes and providing financial support for upfront infrastructure investments:

*“Currently, prescribing digital health apps falls entirely on the shoulders of physicians. I can issue a prescription, but there’s no proper reimbursement—just a few euros, which is meaningless. Online consultations are also poorly compensated; the fees are minimal.”*

*(P06, f, 50–59 years, outpatient private practice, HP)*

Another key facilitating factor identified by the majority of participants concerned the integration of scientific institutions and evidence-based research structures (*n* = 8). Academic networks and professional associations were regarded as pivotal actors in legitimizing and supporting the quality-assured implementation of DHIs. Seven interviewees highlighted the importance of scientific evaluation to ensure DHIs meet ethical and clinical standards. Rigorous validation was considered necessary for guideline-conform usage and measurable treatment outcomes. Follow-up studies were also recommended to assess long-term effects, relapse rates, and sustained utilization over time:

*“The application we’re using has been scientifically evaluated. It has been shown to be effective in treating depression because patients are closely connected to it and can access the system daily. It serves as a direct, rapid, digital support tool.”*

*(P06, f, 50–59 years, outpatient private practice, HP)*

Additionally, four participants emphasized that psychology presents unique implementation challenges compared to somatic medicine, advocating practice-oriented, multiperspective research embedded in clinical institutions:

*“This mainly concerns psychology. Most digital tools are designed for somatic care, and psychology is often neglected. I would like to see more institutional research—talking to staff in psychiatric teams about what they need and what makes sense—rather than just offering tools they then have to figure out how to use on their own.”*

*(P04, f, 50–59 years, outpatient mental health setting, HP*)

Participants also highlighted the role of the Federal Joint Committee (Gemeinsamer Bundesausschuss) as the supreme decision-making body in the German healthcare system. It was suggested to establish an independent platform to collect and disseminate implementation ideas from clinical practice, promoting networking and a bottom-up innovation culture.

Six participants underlined the importance of integrating digital competencies and the use of DHIs into undergraduate and postgraduate medical education. Early exposure to digital tools was considered crucial for fostering both critical digital literacy and confidence in using such tools in clinical settings. The majority recommended embedding DHIs into the curriculum via a foundational module, while a minority favored optional specialization through continuing education and professional development programs:

*“I wouldn’t make it a separate subject. If someone wants to deepen their knowledge, they can do that later. But I do think we need to introduce students to digitalization—during their learning, knowledge acquisition, and in the clinical semesters.”*

*(P06, f, 50–59 years, outpatient private practice, HP)*

A nuanced picture emerged regarding patients’ needs. Several participants reported that younger patients generally demonstrated a positive attitude and greater openness toward engaging with DHIs. High digital competence and frequent engagement with digital media were common in this demographic. In particular, video consultations were widely accepted, as they provide a form of simulated face-to-face interaction, approximating the therapeutic relationship (*n* = 4):

*“Honestly, video consultations are very well received—especially if patients don’t want to or cannot come in. A video consultation is a thousand times better than none at all, and far better than a phone call. Seeing someone simulates a real relationship.”*

*(P05, f, 50–59 years, inpatient mental health setting, HP)*

In contrast, older patients were described as engaging with DHIs less frequently and displaying lower levels of digital competence. Many appeared overwhelmed or skeptical toward using digital technologies, particularly in rural areas. Key barriers cited included limited experience, uncertainty, and fear of making mistakes:

*“We have a wide age range here, and many older people are unfamiliar with these technologies or are hesitant to engage. They didn’t grow up with it and are afraid of making mistakes. I think that’s currently our biggest challenge—that many still don’t know how to use this technology at all.”*

*(P05, f, 50–59 years, inpatient mental health setting, HP)*

Irrespective of digital skills, several participants emphasized intrinsic motivation and critical reflection as essential prerequisites for meaningful adoption and implementation of DHIs (*n* = 3). These factors are influenced by personality traits and clinical diagnoses.

## 4. Discussion

This paper has explored the determinants of organizational implementation based on the CFIR, which are relevant for providing DHIs in depression care. Here, the findings highlight the role of leadership support, the need for facilitators, training, infrastructure, reimbursement, and scientific validation. With regard to the domain individuals, prior experience with DHIs, as well as intrinsic motivation and a support system for potential users, were articulated as essential from the perspective of the interviewed persons. Furthermore, available resources seem to be crucial in the inner setting domain. From the perspective of the interviewees, factors such as user participation and the existence of an implementation strategy are central within the process domain, whereas in the outer setting, legal regulations and scientific evidence play a decisive role. In line with existing work, barriers and facilitators were identified across CFIR-domains, including implementation process and organizational factors, which are often unexplored in the context of technology implementation [31]. HPs emphasize the significance of managerial support, concerns regarding the scientific objectivity of studies evaluating DHI effectiveness, regulatory aspects, concerns regarding the resource-efficient integration of the technologies into workflows, and the integration of relevant HPs in the implementation process. The interviewees reported DHIs can improve the care situation of affected individuals by enabling more targeted, timely, and efficient access to services, which is supported by similar studies [32].

The results indicate that decisions to implement DHIs are shaped by individual goals, intrinsic motivation, and the belief that DHIs benefit professional practice. Various motivations for using DHIs were identified. On the one hand, HPs are intrinsically motivated to use DHIs in order to achieve better results and adequate patient care. On the other hand, the participants expressed that DHIs provide greater professional autonomy and opportunities for tailored patient care, which further motivates their integration into routine practice. In addition, positive experiences with DHIs were associated with successful and sustained implementation, confirming the heterogeneity of individual motives reported in other studies [33,34].

To improve routine care and generate added value for patients, access to relevant information is crucial. Psychotherapists play a central role in this process, yet the results also indicate gaps in their experience and knowledge. An Australian study demonstrated that enhancing knowledge can increase psychotherapists’ acceptance of DHIs [35]. Structured information flows, transparent data protection, and high-quality care are fundamental prerequisites for successful DHI implementation. Interest groups can act as reliable information sources, and strengthening providers’ role in using DHIs may facilitate integration into care. Given high workloads, low affinity for DHIs, and partial skepticism, leveraging established communication channels (professional associations, insurance groups, congresses) could increase awareness of evidence-based quality standards. In the future, the restrictive attitude of HPs is expected to weaken due to demographic changes [36]. However, this effect could be accelerated through a targeted communication strategy.

Participants generally anticipate continued development and increasing integration of DHIs into psychiatric treatment. It is further expected that the range of users will evolve, as increasingly tech-savvy patients with higher digital skills will be treated. Proficient digital skills are necessary for DHIs to be used correctly at all times. Functional problems can otherwise lead to treatment being discontinued, even though these could be easily resolved. For older patients, DHIs may represent an additional burden and stress, especially in acute or critical situations [37]. Technical requirements remain a challenge, especially for patients lacking resources, overwhelmed by technology, or critical of digital tools. Individualized guidance and close initial support are necessary for correct and effective use, highlighting the importance of digital inclusion [38].

From the perspective of HPs, it is imperative to conduct thorough checks of the DHIs’ specifications regarding data security and to ensure the interoperability of data generated by the applications, e.g., for patient records and the providers’ IT systems. The lack of legal guarantees may substantially reduce DHI acceptance [39]. An integrative digital platform for providers must meet high standards of quality assurance, data security, and manageability to offer added value.

In addition to its proven efficacy, effective integration into care represents an important criterion for the utilization of DHIs [40]. Medical ethical guidelines for responsible DHI integration are still debated. Assessing patient (digital) health literacy, for instance, by expanding medical history to include prior DHI use, is necessary to identify gaps [41]. Developing DHIs aligned with patient needs can address ambivalence regarding suitability in different treatment phases.

While bridging waiting times was identified as a suitable phase, respondents were more critical about other phases, emphasizing phase-specific use depending on patient compliance and needs. Integrative approaches, such as blended care, allow efficient allocation of limited therapeutic resources and maintain appropriate patient-therapist contact intensity [42].

To promote DHI implementation in depression care, it is important to address concerns about quality, potential unintended effects, and to increase transparency regarding quality criteria, pricing, and proof of benefit [39,43]. While DHIs can enhance access and efficiency, they should complement rather than replace traditional care. In resource-limited settings, excessive reliance on digital tools may risk reducing personal interaction and care quality. A balanced integration that preserves human contact and clinical judgment remains essential for effective mental health treatment. The involvement of management support and organizational functions plays a crucial role in promoting the implementation of DHIs. Individuals at the management level who are committed to DHIs and experienced in change management within organizational contexts are perceived by service providers as key facilitators of implementation. These findings highlight the importance of motivated and knowledgeable individuals for the implementation process. However, implementation can become successful if the process is well planned and there are clearly defined roles and responsibilities for the management and potential users. A clearly defined and communicated strategy has the potential to increase stakeholder awareness, namely among end-users of DHIs, and consequently enhance user satisfaction. This is in line with the findings of a study exploring the necessity of extending traditional roles and conditions [44] as well as stable organizational management [45]. The present study further indicates that adequate staffing resources, targeted support and training, organizational commitment, and the integration of DHIs into existing care structures are critical for the implementation of DHIs in specialized settings like psychiatric institutions. It is important to test the adaptability of the technology and improve it with a user-centered approach in design and implementation. The implementation involves interdisciplinary staff engagement, clear communication, and continuous assessment of implementation requirements and conditions at the management level [46]. Staff engagement can be facilitated by providing information in advance to improve efficiency and effectiveness, accommodating different needs by offering multiple ways to give feedback. The presented results may guide implementation leaders towards sustainable and patient-centered introduction of DHIs in depression care.

The interviewees attached great importance to feedback from patients, especially from their own practice, regarding their experiences with DHIs. Currently, HPs’ DHI use depends on patient engagement, which only occurs once patients use DHIs. Since patients rely on HPs as information sources [47], providing training enables providers to identify useful DHIs and support their integration. Successful DHI use requires meaningful integration into existing care pathways, appropriate technical and time resources, and adequately funded further training. For this reason, there may be a need for an independent accredited curriculum for future practitioners, alongside further training for existing staff. Such programs should be targeted to the specific audience to ensure adequate service provision. Allowing experienced persons, such as trained practitioners as well as people familiar with DHI, to support the guidance of DHI may also aid in alleviating barriers around the capacity to implement interventions.

Overall, this study focused on identifying factors influencing the implementation of DHIs from the perspective of relevant stakeholders, using the CFIR. This study extends the existing literature on the implementation of DHI for depression care in terms of focusing on organizational conditions and highlighting the role of organizational support and implementation facilitators from the perspective of potential users. While most factors were assessed as independent of each other, several were also described as interacting. For example, access to expert evidence may have an influence on the intrinsic motivation to use DHIs in one’s own work routine. Like existing studies [31,48], these results show the dynamic relationships between individual factors. Understanding these interrelationships is therefore crucial for the successful implementation of DHIs.

The explorative approach to the integration of different service providers proved useful, as the overlapping concerns of the interest groups provide important starting points for implementation strategies. The semi-structured approach was critical in uncovering additional CFIR-related determinants that warrant consideration due to their relevance within the implementation context. Nevertheless, the results should be interpreted in light of certain limitations. First, the achieved sample size of service providers was smaller than anticipated. In addition, potential bias in the findings should be considered, for example, due to the regional concentration of respondents. For future research, it would be useful to include a more heterogeneous sample to increase contrast between participants. Furthermore, selection bias may have occurred, as participants were likely more open and motivated toward DHI implementation than the broader population of service providers. Response bias may also have influenced findings, potentially leading to more positive assessments of implementation than would be expected from a representative sample. Additionally, the present study is based on a sample of nine HPs, which limits the generalizability of the results. Despite employing diverse strategies, recruitment remained challenging. Furthermore, the gender distribution is unbalanced (eight women, one man). While this partly reflects the feminized professional structure in the field of psychological psychotherapy—according to the Federal Chamber of Psychotherapists, approximately 73% of licensed psychotherapists are female [49]—a potential influence of gender-specific perspectives on the results cannot be ruled out.

Further, it should be noted that the CFIR applied in this study represents a customized version. During the research process, the framework’s domains were adapted to better reflect the study’s specific focus and context. A notable limitation is the exclusion of the CFIR “intervention” domain. While this decision was made pragmatically to focus on organizational factors and reduce interview length, it creates a conceptual gap. However, several constructs belonging to the Intervention domain—such as adaptability, usability, cost, and perceived evidence strength—emerged naturally within the data and were analyzed inductively within the other CFIR domains. Therefore, while the analysis was not fully CFIR-based, it can be considered CFIR-informed, combining deductive framework guidance with inductive openness to emergent intervention-related factors. This approach allowed the study to capture key implementation determinants relevant to the intervention domain, while maintaining analytical depth within the organizational focus. Intervention characteristics often interact with organizational conditions, influencing uptake and implementation success. As a result, certain DHI-specific barriers or facilitators—such as usability, design features, or integration with clinical workflows—may not have been fully captured. Future studies should include the intervention domain to explore these interactions and provide a more comprehensive understanding of implementation determinants.

The modified framework thus served as a theory-informed analytical tool, aligned with the study’s aim of examining structural and contextual factors influencing the implementation of DHI in depression care.

Moreover, the qualitative results reflect individual responses from employees and cannot be generalized due to the explorative approach. Qualitative research generally seeks to explore in-depth experiences and perspectives of a specific group or population, rather than seeking to generalize findings to larger populations. However, this can pose challenges in translational health research, where policymakers and healthcare providers may need information that is generalizable to make informed decisions.

Due to its nature, qualitative data are not qualified for statistical analysis but are valuable for evaluating feasibility and capturing user perspectives. Future research should aim for a larger and more diverse sample, including broader regional representation and a balanced gender distribution, to reduce potential selection and response biases.

## 5. Conclusions

The empirical analysis of HPs’ perspectives on the challenges, potentials, and expectations associated with DHIs in depression treatment highlights key areas requiring further action to support successful implementation. At the same time, it provides valuable insights into strategic approaches that can enhance patient care. Open and transparent communication among all stakeholders involved in depression care is essential to ensure a shared and comprehensive understanding of DHIs. Such communication reduces uncertainties and mitigates perceived gaps in digital competence.

The goal of implementing DHIs is not to digitalize or standardize all aspects of care, but to use them as complementary tools integrated meaningfully into routine practice. Users should be sensitive to the potential concerns of DHIs, such as excessive reliance on DHIs. This overreliance could lead to habituation effects and overshadow human expertise—especially among younger staff, who may rely too heavily on DHIs. However, when implemented under appropriate conditions, DHIs have the potential to optimize the use of available therapeutic resources and contribute to closing the existing treatment gap in mental healthcare.

To facilitate effective integration of DHIs, tailored training programs and structured support for HPs should be established. Implementation strategies must be context-sensitive and co-developed with end-users to ensure both acceptance and sustainability. Further research should examine setting-specific barriers and facilitators, as well as the long-term impact of DHIs on clinical outcomes and care processes. In essence, the study accentuates the need for tailored implementation and training strategies as well as solid evaluation methods with regard to scientific evidence and effectiveness. This study’s insights offer guidance for future endeavors, propelling DHI into integral components of depression care approaches.

## Figures and Tables

**Table 1:** Characteristics of participating health professionals (*n* = 9).

Participant ID	Sex	Age Ranges	Profession	Affinity of Technology
01	Female	40–49	Advanced practice psychiatric nurse	2.8
02	Female	21–29	Specialist in psychiatry	4.4
03	Female	30–39	Specialist in psychotherapy	3.3
04	Female	50–59	Specialist in psychotherapy	2.4
05	Female	50–59	Medical and psychological psychotherapist	2.8
06	Male	50–59	General practitioner	4.1
07	Not disclosed	Not disclosed	Specialist in psychotherapy	4
08	Female	40–49	Medical and psychological psychotherapist	1.8
09	Female	30–39	Specialist in psychotherapy	3.2

## Data Availability

Due to privacy and ethical restrictions, the raw interview data are not publicly available. Access will be provided to academic researchers who agree to maintain participant confidentiality and use the data exclusively for scholarly purposes.

## Supplementary Materials

The following supporting information can be downloaded at: https://www.mdpi.com/article/10.3390/healthcare13212717/s1, Supplementary Material S1: Standards for Reporting Qualitative Research; Supplementary Material S2: Semi-structured Interview Guide; Supplementary Material S3: Affinity for Technology Questionnaire; Table S1: CFIR-informed Category System; and Table S2: Analytical Codebook.

## Abbreviations

The following abbreviations are used in this manuscript:

ATI	Affinity for Technology Interaction
CFIR	Consolidated Framework for Implementation Research
DHI	Digital Health Intervention
HP	Health Professionals
