# Supplementary material for: Implementation Factors of Digital Health Interventions in Depression Care—The Perspective of Health Professionals
Source: Healthcare (Basel). 2025 Oct 27;13(21):2717. doi: 10.3390/healthcare13212717 (PMC12608834; doi:10.3390/healthcare13212717)
Supplement: Supplementary file 1 [file healthcare-13-02717-s001.zip › Supplementary S2 Semi-structured Interview Guide.pdf]

Supplementary Material S2 Semi-structured interview guide for health professionals

Deutsch	English Translation
Die Teilnehmenden wurden über Ziel, Ablauf und Vertraulichkeit informiert. Das Interview dauerte ca. 30–45 Minuten. Eine schriftliche Einverständniserklärung wurde eingeholt.	Participants were informed about the purpose, procedure, and confidentiality. Each interview lasted approximately 30–45 minutes. Written informed consent was obtained prior to participation.

A. Allgemeine Einstiegsfragen / General Introductory Questions

Deutsch	English Translation
Was verstehen Sie unter „digitaler Gesundheitstechnologie"?	What do you understand by "digital health technology"?
Welche digitalen Technologien nutzen Sie bereits in Ihrem beruflichen Alltag?	Which digital applications or technologies do you currently use in your professional practice?
Welche Erfahrungen haben Sie bisher mit digitalen Gesundheitstechnologien gemacht?	What experiences have you had so far with digital health applications?

B. Inner Setting – Organisationsbezogene Faktoren / Organizational Factors

Deutsch	English Translation
Sehen Sie grundsätzlich die Notwendigkeit, digitale Gesundheitstechnologien in Ihrer Einrichtung zu implementieren? Warum (nicht)?	Do you see a general need to implement digital health technologies in your organization? Why or why not?
Wer sollte aus Ihrer Sicht für die Implementierung verantwortlich sein?	Who do you think should be responsible for the implementation?
Wie sollte die Implementierung intern kommuniziert werden?	How should the implementation be communicated within the team?
Sollten alle Teammitglieder in den Entscheidungsprozess eingebunden werden?	Should all team members be involved in the decision-making process?
Inwiefern passt der Einsatz digitaler Gesundheitsechnologien zur Mission oder Philosophie Ihrer Einrichtung oder Praxis?	To what extent does the use of digital technologies align with your institution's or practice's mission or philosophy?
Welche Anreize wären aus Ihrer Sicht wichtig, um die Implementierung zu fördern (z. B. Zeit, Anerkennung, Schulung)?	What incentives do you consider important to support implementation (e.g., time, recognition, training)?

Deutsch	English Translation
Welche Rolle spielt die Leitung bei der Einführung digitaler Gesundheitstechnologien?	What role should leadership play in the introduction of digital health applications?
Wie sollten Informationen zu digitalen Gesundheitstechnologien bereitgestellt werden (z. B. durch Experten, Kollegen, Materialien)?	How should information about digital health applications be provided (e.g., by experts, colleagues, written materials)?

C. Process – Implementierungsprozess / Implementation Process

Deutsch	English Translation
Welche organisatorischen Aspekte sollten bei der Planung berücksichtigt werden?	Which organizational aspects should be considered during the planning phase?
Welche Herausforderungen sehen Sie im Implementierungsprozess?	What challenges do you see during the implementation process?
Welche Maßnahmen sind aus Ihrer Sicht wichtig, um die Implementierung zu reflektieren oder zu evaluieren?	What do you consider important for reflecting on or evaluating the implementation process?
Inwiefern kann eine Pilotphase hilfreich sein, um Akzeptanz und Wirksamkeit zu prüfen?	To what extent can a pilot phase help to test acceptance and effectiveness?
Wie könnte eine kontinuierliche Bewertung und Anpassung während der Implementierung aussehen?	What might continuous assessment and adaptation during implementation look like?

D. Outer Setting – Externe Rahmenbedingungen / External Environment

Deutsch	English Translation
Welche Bedeutung haben politische, gesellschaftliche oder wirtschaftliche Rahmenbedingungen für die Implementierung digitaler Gesundheitstechnologien?	What significance do political, societal, or economic conditions have for the implementation of digital health applications?
Welche Rolle spielt die Gesundheitspolitik bei der Integration digitaler Gesundheitstechnologien in die Regelversorgung?	What role does health policy play in integrating digital applications into routine care?
Welche Gesetze oder Regulierungen beeinflussen die Implementierung?	Which laws or regulations influence the implementation?

Deutsch	English Translation
Welche Rolle spielt die Gesellschaft bei der Akzeptanz und Nutzung digitaler Gesundheitstechnologien?	What role does society play in the acceptance and use of digital health applications?
Gibt es einen Druck von außen, z. B. durch andere Einrichtungen oder Berufsgruppen?	Is there any external pressure, e.g., from other institutions or professional groups?
Welche externen Partner sind wichtig für die Implementierung?	Which external partners are important for implementation?
Welche finanziellen oder strukturellen Fördermöglichkeiten könnten hilfreich sein?	Which financial or structural incentives could support implementation?

Abschluss / Closing

Deutsch	English Translation
Gibt es noch etwas, das Sie ergänzen möchten oder das bisher nicht angesprochen wurde?	Is there anything you would like to add or that has not yet been discussed?
Wenn Sie nach dem Gespräch noch weitere Gedanken haben, können Sie diese gerne nachreichen.	If you have additional thoughts after the interview, you are welcome to share them later.
