# Supplementary material for: Implementation Factors of Digital Health Interventions in Depression Care—The Perspective of Health Professionals
Source: Healthcare (Basel). 2025 Oct 27;13(21):2717. doi: 10.3390/healthcare13212717 (PMC12608834; doi:10.3390/healthcare13212717)
Supplement: Supplementary file 1 [file healthcare-13-02717-s001.zip › Supplementary S3 ATI (Questionnaire Affinity for Technology).pdf]

Affinity for Technology Interaction (ATI) Scale

Franke, Attig, & Wessel (2019)

In the following questionnaire, we will ask you about your interaction with technical systems. The term "technical systems" refers to apps and other software applications, as well as entire digital devices (e.g., mobile phone, computer, TV, car navigation).

Please indicate the degree to which you agree/disagree with the following statements.		completely disagree	largely disagree	slightly disagree	slightly agree	largely agree	completely agree
01	I like to occupy myself in greater detail with technical systems.	☐	☐	☐	☐	☐	☐
02	I like testing the functions of new technical systems.	☐	☐	☐	☐	☐	☐
03	I predominantly deal with technical systems because I have to.	☐	☐	☐	☐	☐	☐
04	When I have a new technical system in front of me, I try it out intensively.	☐	☐	☐	☐	☐	☐
05	I enjoy spending time becoming acquainted with a new technical system.	☐	☐	☐	☐	☐	☐
06	It is enough for me that a technical system works; I don't care how or why.	☐	☐	☐	☐	☐	☐
07	I try to understand how a technical system exactly works.	☐	☐	☐	☐	☐	☐
08	It is enough for me to know the basic functions of a technical system.	☐	☐	☐	☐	☐	☐
09	I try to make full use of the capabilities of a technical system.	☐	☐	☐	☐	☐	☐

Analysis

1. When entering the participants' responses into a data file for the analysis, the responses should be coded as follows: completely disagree = 1, largely disagree = 2, slightly disagree = 3, slightly agree = 4, largely agree = 5, completely agree = 6.
2. Responses to the **three negatively worded items** (items 3, 6, 8) **need to be reversed** (6=1, 5=2, 4=3, 3=4, 2=5, 1=6).
3. Finally, a mean score should be computed over all 9 items.
4. Report mean (*M*), standard deviation (*SD*) and Cronbach's alpha, usually with two decimal places, e.g., *M* = 3.61, SD = 1.08, α = .87.

Source: Franke, T., Attig, C., & Wessel, D. (2019). A Personal Resource for Technology Interaction: Development and Validation of the Affinity for Technology Interaction (ATI) Scale. *International Journal of Human–Computer Interaction, 35*(6), 456-467, DOI: 10.1080/10447318.2018.1456150
